# Effects of forefoot vs. rearfoot landing on biomechanical risk factors for lower limb injuries and performance during stop-jumping tasks

**DOI:** 10.3389/fspor.2025.1676448

**Published:** 2025-11-18

**Authors:** Tianchen Huang, Yuqi He, Lizhi Mao, Mianfang Ruan, Daisuke Takeshita

**Affiliations:** 1 Department of Life Sciences, Graduate School of Arts and Sciences, The University of Tokyo, Tokyo, Japan; 2 Faculty of Sports Science, Huaqiao University, Quanzhou, China; 3 College of Physical Education and Health, Wenzhou University, Wenzhou, China

**Keywords:** stop-jump task, landing strategies, forefoot landing, rearfoot landing, ACL injuries, ankle sprain, jump performance

## Abstract

**Introduction:**

Lower limb injuries commonly occur during sudden deceleration movements, where landing technique plays a critical role. The choice between forefoot and rearfoot landing strategies remains debatable, especially when considering both injury prevention and performance optimization. This study aimed to evaluate the effect of the forefoot and rearfoot landing on lower limb biomechanical risk factors and performance during a stop-jumping task.

**Methods:**

Twenty-three healthy male subjects performed a stop-jumping task under forefoot and rearfoot landing conditions, during which 3D kinematic, kinetic, and performance data were collected.

**Results:**

Forefoot landing exhibited significantly greater ankle plantarﬂexion (−26.02° vs. 18.81°) and internal rotation (3.67° vs. −3.32°) at initial contact compared to rearfoot landing (*p* < 0.001). In the early landing phase, forefoot landing demonstrated larger posterior ground reaction force (GRF) (0%–41%), more posteriorly inclined GRF angle (0%–20%), and more vertical inclined shank angle (11%–57%). Hip ﬂexion angles (20%–91.3%) and abduction angles (27.9%–98.5%) were smaller and the knee abduction angles (16.4%–28.2%) were greater in forefoot. Forefoot landing achieved shorter stance time (396.75 vs. 433.48 ms, *p* = 0.01) while maintaining similar jump height (49.51 vs. 50.07 cm, *p* = 0.34) compared to rearfoot landing.

**Discussion:**

Forefoot landing during stop-jumping tasks demonstrated distinct biomechanical patterns including increased posterior GRF and more posteriorly inclined GRF angle during early stance, potentially reducing anterior cruciate ligament loading while providing performance benefits through decreased stance time without compromising jump height. However, the increased ankle internal rotation observed during forefoot landing suggests an elevated risk of lateral ankle sprain. These findings suggest a potential trade-off between knee protection and ankle vulnerability that should be considered when developing landing technique training programs.

## 1 Introduction

Lower limb injuries are a major concern in competitive sports, with knee (29.3%) and ankle (22.4%) injuries being the most prevalent (1). Non-contact anterior cruciate ligament injuries (NACLIs) account for approximately 70% of all ACL injuries (2, 3) and can lead to long-term consequences such as impaired performance, high risk of re-injury, and osteoarthritis (4, 5). Similarly, lateral ankle sprains also represent a prevalent and clinically significant injury (6, 7). These injuries can result in persistent joint dysfunction and elevated long-term health risks (8, 9). Despite the commonly reported higher ACL injury rates in female athletes (10, 11), a study based on twenty-one years of data found that recreational males accounted for more total injuries (12), yet remain underrepresented in current researches. Understanding the biomechanical mechanisms underlying these injuries and developing effective prevention strategies through movement analysis in recreational males are critical priorities in sports biomechanics.

Many studies have evaluated landing techniques as strategies to reduce injury risk factors (13–16). However, there is debate over which biomechanical planes are most relevant. Some argue that sagittal plane data alone cannot fully explain ACL loading, emphasizing the need for three-dimensional analysis (17–19). Others highlight sagittal-plane factors such as anterior shear force or axial compression as primary contributors (20–22). Together, these findings suggest that understanding how landing strategies inﬂuence joint loading across all planes is essential.

Stop-jumping maneuvers, involving rapid deceleration followed by vertical jumping, are common in sports such as basketball and volleyball and pose a high risk of lower limb injuries (23). Two primary landing styles are employed: forefoot and rearfoot. Video analyses show that many ACL injuries occur following rearfoot or ﬂatfooted landings (24). Boden et al. (20) advocated for instructing athletes to adopt a forefoot landing strategy as a means to reduce the risk of ACL injury. However, before establishing more specific injury prevention strategies, it is essential to clarify the biomechanical differences between forefoot and rearfoot landings, as well as their effects on athletic performance. Cortes et al. (25) reported that during sidestep cutting and pivot tasks, female athletes exhibited increased knee adduction moments when employing a forefoot landing pattern. Zhou et al. (16) found that forefoot landing during stop-jumping tasks was associated with increased knee ﬂexion angles. Similarly, Uno et al. (26) observed similar results during cutting maneuvers. Rearfoot landings may limit calf muscle engagement, reducing shock absorption and increasing knee loading. Conversely, forefoot landings may enhance force absorption and jump performance via improved stretch-shortening cycle use (27, 28) but may elevate ankle joint loading and lateral ankle sprain risk. Rearfoot landing may reduce some ankle-related risks while increasing knee load and impairing performance. Although prior studies show landing technique inﬂuences knee mechanics (25, 26), considering that most sports injuries occur during moments of high performance demand (29, 30), the lack of analysis on athletic performance may undermine the reliability of the evidence.

In sports biomechanics, balancing injury prevention with performance optimization remains a critical yet underexplored challenge. While many studies examine injury risk, few assess how landing strategies also affect performance, especially in demanding tasks like stop-jumping tasks where both joint loading and outcomes such as jump height are crucial (31). The effects of forefoot vs. rearfoot landing on both injury-related knee and ankle mechanics and performance metrics remain unclear. Moreover, most research addresses either knee or ankle joints in isolation, neglecting the integrated lower limb response. To address these gaps, this study investigated how forefoot and rearfoot landing strategies during stop-jumping maneuvers affect: (1) knee biomechanics related to ACL injury, (2) ankle biomechanics related to lateral ankle sprain risk, and (3) performance outcomes including jump height and stance time.

Based on existing literature, we hypothesized that in recreational males, compared to rearfoot landing: (1) Forefoot landing would reduce ACL injury risk factors including peak knee valgus angle, knee extension moment, and anterior tibial shear force, (2) Forefoot landing would increase ankle injury risk factors including peak ankle inversion angle and inversion moment, and (3) Forefoot landing would enhance jump height and reduce stance time through more effective utilization of the stretch-shortening cycle.

## 2 Method

### 2.1 Participants

The experimental procedure was approved by the University of Tokyo Ethics Committee. Twenty-three healthy, recreational active males were recruited for this study (age: 26.5 ± 8.7 years; height: 1.78 ± 0.05 m; weight: 74.84 ± 10.21 kg). Each participant provided written informed consent. To ensure participant standardization, inclusion criteria included the following: (1) no history of lower limb surgical procedures; (2) regular engagement in recreational physical activity at least three times per week, with each session lasting a minimum of 30 min. Exclusion criteria included: (1) any current or previous lower limb injury (including ligament, tendon, or joint pathologies) within the past 12 months, regardless of whether surgery was performed; (2) history of neurological, cardiovascular, or balance disorders that could affect motor performance; and (3) inability to perform the stop-jump task safely.

### 2.2 Experimental protocol

**Figure 1:**
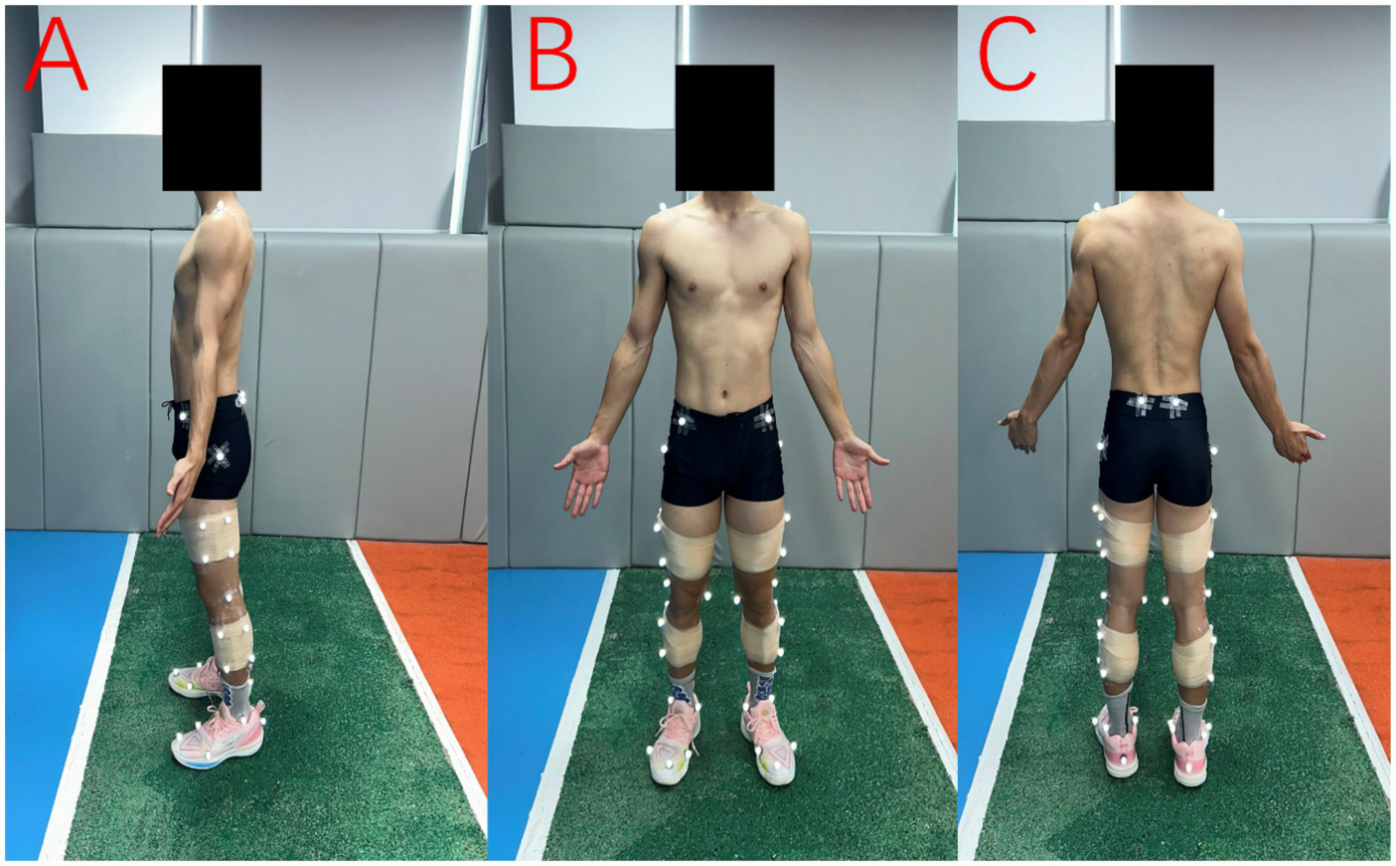
Retroreﬂective marker placement shown from three views: **(A)** sagittal, **(B)** anterior, and **(C)** posterior. Markers were placed bilaterally on anatomical landmarks, with rigid marker clusters attached to the thighs and shanks using neoprene straps.

Participants were asked to warm up for five minutes in their own manner, which generally consisted of running and stretching. As all participants were right-leg dominant, which was determined based on the preferred jumping leg in a single leg jump for distance, the experiment and data analyses were conducted on the right leg. All participants wore identical models of spandex shorts and shoes, sized appropriately for each participant, as required for the formal experiment. Forty retroreﬂective markers were placed bilaterally on the acromioclavicular joints, anterior superior iliac spines, posterior superior iliac spines, greater trochanters, medial and lateral femoral epicondyles, medial and lateral malleoli, and first and fifth metatarsophalangeal joints, first toes, and heels (Figure 1). Clusters of four non-collinear markers mounted on rigid thermoplastic shells were attached to the lateral sides of both thighs and shanks using neoprene straps (32).

**Figure 2:**
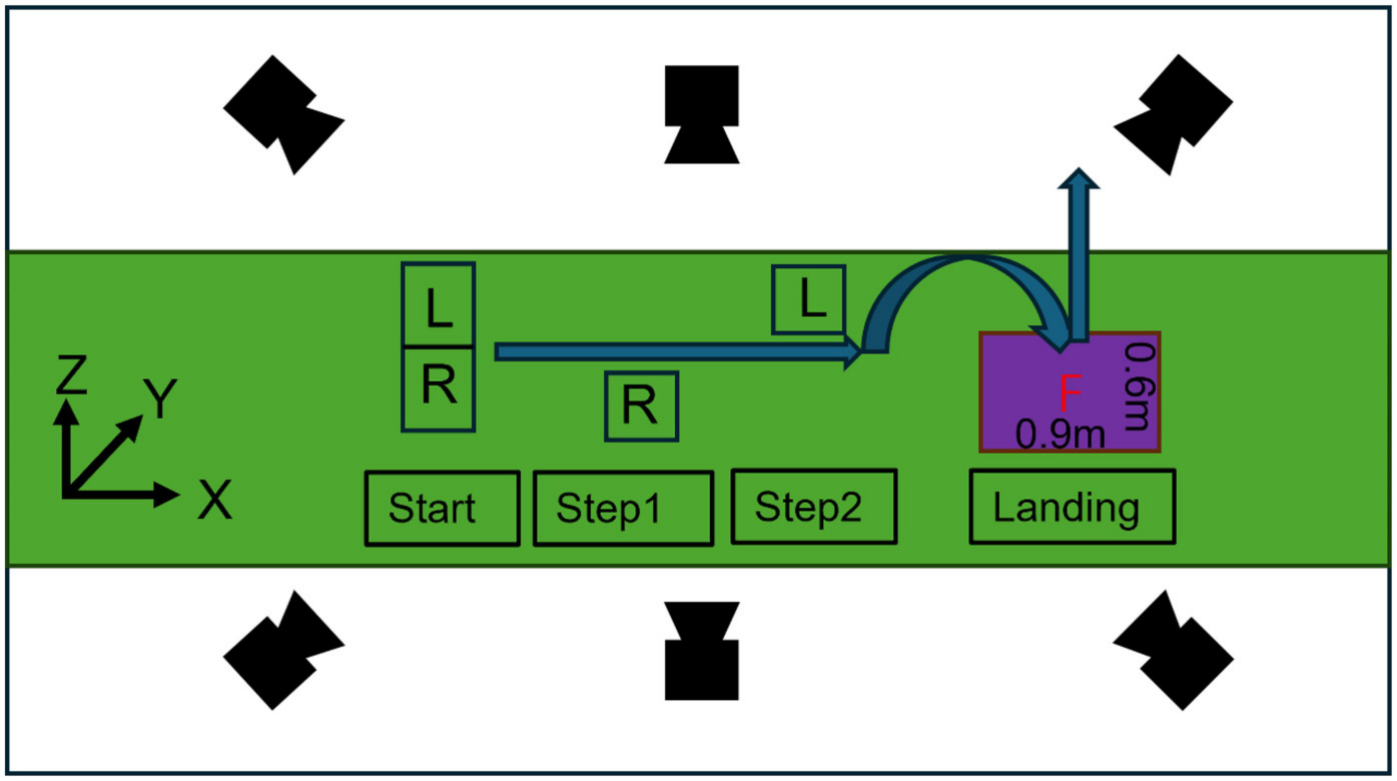
Experimental setup for the stop-jump task. The global coordinate system is defined as: X (anterior+), Y (lateral+), Z (vertical+). The approach path includes start position, and two stepping positions (Step1, Step2) marked for foot placement (L: left foot; R: right foot), leading to a 0.9 m × 0.6 m force platform for the landing phase.

**Figure 3:**
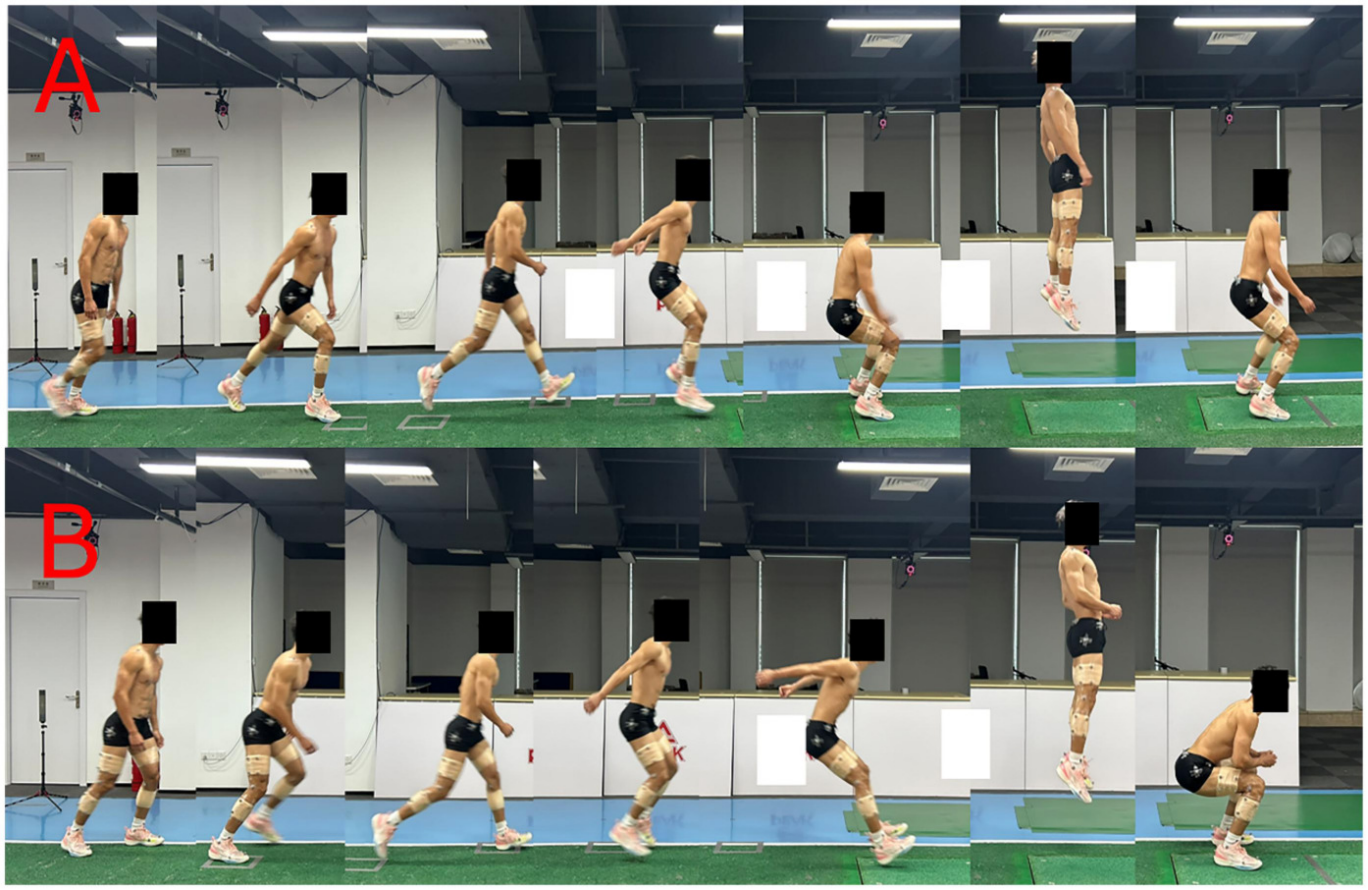
Stop-jump task sequences showing **(A)** forefoot landing and **(B)** rearfoot landing techniques. Each sequence illustrates the approach run, landing phase, and vertical jump.

After completing marker placement, a three-second static calibration trial was conducted on the force plate. Following static calibration, the six anatomical markers on the medial sides of both knees, ankles, and feet were removed. The stop-jumping task consisted of an approach run, both-foot landing, and a vertical jump. The participants were instructed to complete the movement as quickly as possible and jump as high as they could. The experimental setup is shown in Figure 2. Five successful stop-jumping trials were required in this study. A trial was considered successful when the following criteria were met:

1. The participant completed the approach run and landed stably with both feet on the force platform, without stepping, sliding, or exhibiting any noticeable loss of balance; (2) The right foot fully contacted the force platform upon landing and remained entirely within its boundaries throughout the stance phase;
2. The participant performed an immediate vertical jump after landing, without any forward or lateral deviation, and clearly left the ground during take-off; (4) The ground reaction force signals were continuous and free from missing data or external interference; and (5) The approach, braking, and jumping actions followed the task instruction—running in as fast as possible, stopping abruptly, and then jumping vertically. Trials that involved any foot stepping off the platform, loss of balance, non-vertical take-off, or incomplete force data were deemed invalid and repeated until five successful trials were obtained. As shown in Figure 3, the 2 experimental conditions for the stop-jumping task were (1) forefoot landing; (2) rearfoot landing at initial ground contact. Task sequences and conditions were individually randomized for each participant using a custom MATLAB script. There was 2-minute rest between conditions and a 30-second rest between trials to avoid any fatigue.

### 2.3 Data collection

A motion capture system consisting of thirteen Mars 4H cameras, 4.1-megapixel resolution (NOKOV Motion Capture System, Beijing, China), was used to collect raw marker coordinate data at 240 Hz. A force platform (1,200 Hz, 9287CA, Kistler Instruments, Winterthur, Switzerland) was placed below ground level in the middle of the runway for ground reaction force data collection. These two data collection systems were synchronized.

### 2.4 Data reduction and processing

The video recordings of the retroreﬂective marker trajectories were digitized using the NOKOV Seeker video analysis system. For each participant, the five trials of each stop-jumping task condition were digitized, starting from 10 frames before right foot initial ground contact to 10 frames after takeoff. To maintain consistency in filtering between kinematic and kinetic data, we applied a low-pass filter with the same cut-off frequency of 6 Hz to both ground reaction force and marker coordinate data (33). Force-based gait events were used to obtain the time-normalized landing phase. The ground contact phase was defined as the duration from initial contact to takeoff during the first landing of the stop-jumping task. Initial contact and takeoff were determined using a vertical ground reaction force (GRF) threshold of 10 N (16, 34). The duration of landing phase was scaled to 101 data points. Visual3D biomechanical software (C-Motion, Germantown, MD, USA) was used to create a kinematic model made of seven skeletal segments (pelvis, bilateral thighs, shanks, and feet) from the standing calibration trial. The pelvis angle was defined as the orientation of the pelvis relative to the laboratory coordinate system, with 0° corresponding to the alignment of the pelvis coordinate system with the laboratory coordinate system. The knee joint angle was defined as 0° when the thigh segment coordinate system was aligned with the shank segment coordinate. The ankle joint angle was defined as 0° in the standing trial. The three-dimensional joint kinematics were calculated using an XYZ Cardan sequence of rotations, with X representing ﬂexion-extension, Y representing abduction-adduction, and Z representing internal-external rotation (35). Shank and GRF inclination angle are relative to the global vertical axis in the sagittal plane. Positive values indicate anterior inclination (forward tilt), while negative values represent posterior inclination (backward tilt). The shank inclination angle was measured between the shank segment's longitudinal axis and the vertical axis, while the GRF inclination angle was measured between the GRF vector and the vertical axis, as defined by Uno et al. (26). The joint moments were estimated using the inverse dynamics approach (36). We normalized the joint resultant forces and moments by body weight. Stance time was defined as the duration between the initial rise and final drop of the vertical ground reaction force corresponding to right foot contact and toe-off events. Each participant was represented by the ensemble average of five successful trials for each foot-strike condition.

### 2.5 Statistical analysis

Paired *t*-tests were used to evaluate the differences in kinematic and kinetic variables across various stop-jumping strategies. Statistical Parametric Mapping (SPM) analysis with paired *t*-tests was used to compare kinematic and kinetic time-series data across the stance phase. A custom MATLAB script was used to interpolate the data points into a time series curve consisting of 101 points, representing 0% to 100% of the landing phase. The statistical analysis was performed using the open-source SPM1d script for paired-sample *t*-tests to analyze the difference in kinematics and kinetics data during landing phase, with the significance level set at 0.05 (37).

## 3 Results

### 3.1 Joint angles in three planes (sagittal, frontal, transverse)

**Figure 4:**
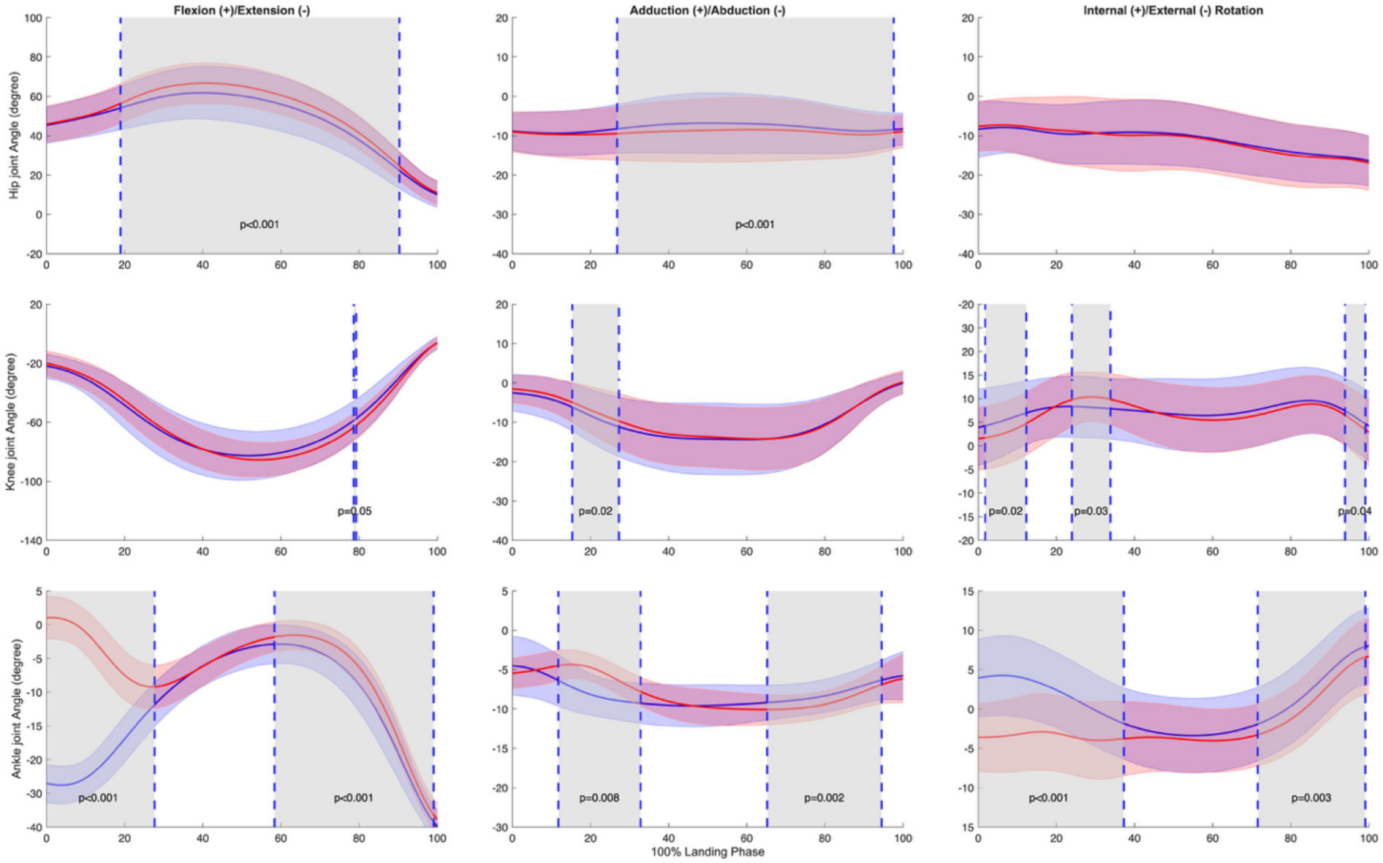
Mean ± SD and SPM t-values of hip, knee, and ankle joint angles between forefoot landing (blue) and rearfoot landing (red) in three planes (sagittal, frontal, transverse) from initial ground contact to takeoff (normalized to 100% landing phase) in 23 subjects. The significance level was set as *p* = 0.05. Statistical differences are highlighted in grey-shaded regions, indicating *p* < 0.05.

Three-dimensional joint kinematic analysis revealed distinct patterns between landing strategies (Figure 4). In the saggittal plane, forefoot landing exhibited significantly reduced hip ﬂexion throughout the majority of the landing phase (20.0%–91.3%, *p* < 0.001, *d* = –0.715). At the knee, a brief interval of increased ﬂexion was observed in the forefoot condition (79.7%–80.2%, *p* = 0.0500, *d* = 0.623). The ankle demonstrated the most pronounced differences, with forefoot landing producing substantially greater plantarﬂexion during early stance (0.0%–28.6%, *p* < 0.001, d = –6.874) and sustained reduced dorsiﬂexion into late stance (59.4%–100.0%, *p* < 0.001, *d* = –0.947).

In the frontal plane, forefoot landing shows a prolonged decrease in abduction angle was observed during 27.9%–98.5% of the landing phase (*p* < 0.001, *d* = 0.982). The knee exhibited significantly increased abduction under forefoot conditions during 16.4%–28.2% of stance (*p* = 0.0174, *d* = –0.791). At the ankle, two intervals of significant difference emerged: forefoot landing showed greater eversion from 12.8%–33.8% (*p* = 0.0083, *d* = –1.374), followed by smaller between 66.3%–95.4% of the landing phase (*p* = 0.0015, *d* = 0.834).

In the transverse plane, significant differences were observed at the knee and ankle, while the hip showed no condition-dependent variation. The knee joint exhibited three discrete intervals of altered rotation: greater internal rotation in forefoot landing during early stance (2.8%–13.3%, *p* = 0.0238, *d* = 0.737) and terminal stance (94.9%–100.0%, *p* = 0.0419, *d* = 0.813), along with increased internal rotation during mid-stance under rearfoot condition (25.0%–34.8%, *p* = 0.0262, *d* = –0.847). At the ankle, forefoot landing induced significantly greater internal rotation throughout early stance (0.0%–38.2%, *p* < 0.001, d = 2.231) and again in late stance (72.6%–100.0%, *p* = 0.0028, *d* = 0.665).

### 3.2 Joint moments in three planes (sagittal, frontal, transverse)

**Figure 5:**
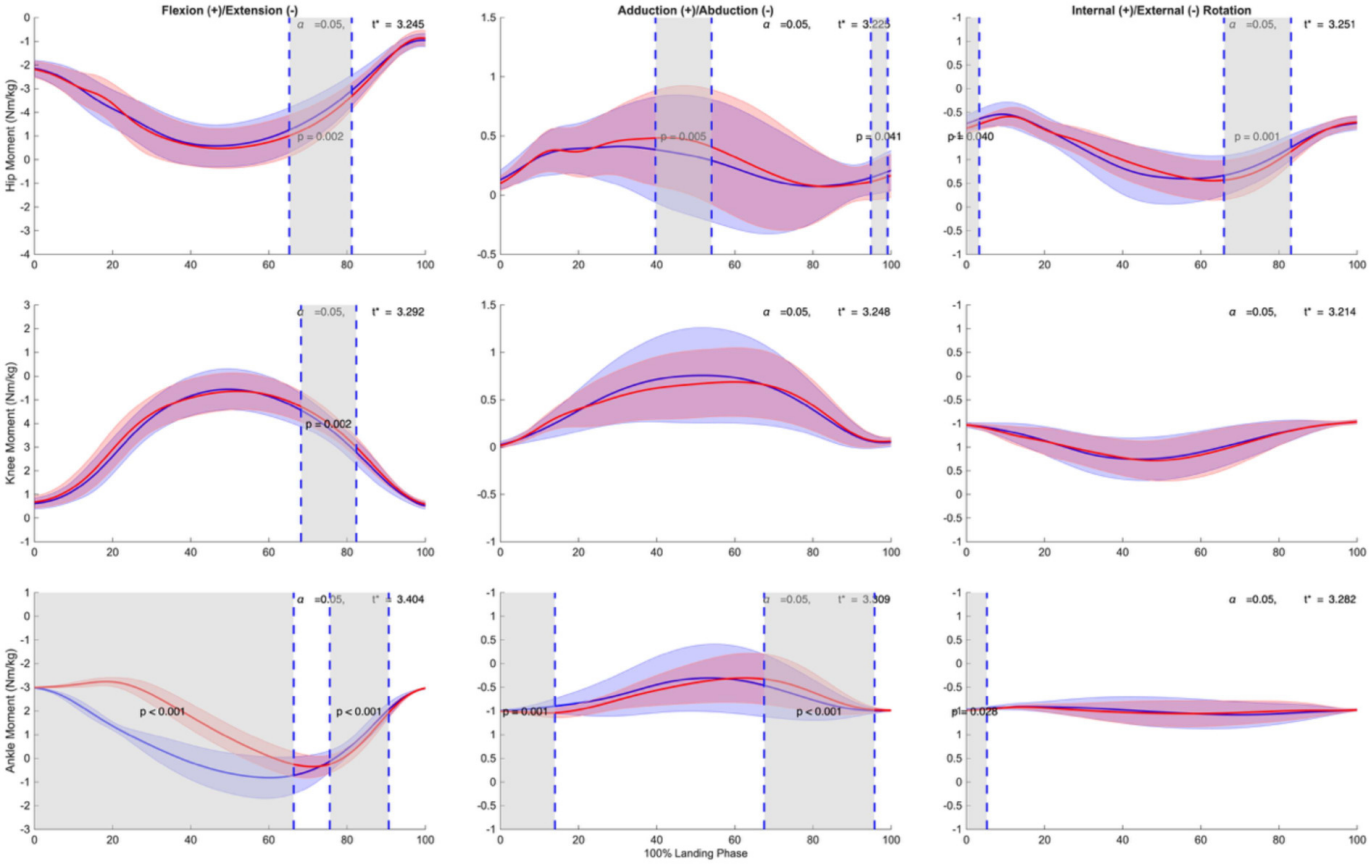
Mean ± SD and SPM t-values of hip, knee, and ankle joint moments comparing forefoot landing (blue) and rearfoot landing (red) in three planes (sagittal, frontal, transverse) from initial ground contact to takeoff (normalized to 100% landing phase) in 23 subjects. The significant level was set as *p* = 0.05. Statistical differences are highlighted in grey-shaded regions, indicating *p* < 0.05.

Joint moment analysis revealed significant differences between landing strategies (Figure 5). In the sagittal plane, forefoot landing was associated with decreased hip ﬂexion moment during late stance (66.2%–82.1%, *p* = 0.0021, *d* = 0.882). The knee showed reduced ﬂexion moment under forefoot conditions in a similar phase window (69.3%–83.3%, *p* = 0.0022, *d* = –0.797). The ankle exhibited pronounced modulation, with significantly greater plantarﬂexion in forefoot landing throughout early to mid-stance (0.5%–67.3%, *p* < 0.001, *d* = –3.802), followed by a brief increase in dorsiﬂexion during late stance (76.6%–91.6%, *p* < 0.001, *d* = 1.466).

In the frontal plane, the hip joint showed reduced adduction in forefoot landing during mid-stance (40.7%–55.0%, *p* = 0.0051, *d* = –0.789), followed by increased adduction during terminal stance (95.9%–100.0%, *p* = 0.0413, *d* = 0.765). No significant differences were detected at the knee. At the ankle, forefoot landing exhibited greater inversion in early stance (0.0%–14.9%, *p* = 0.0011, *d* = 0.726) and reduced inversion during late stance (68.5%–96.7%, *p* < 0.001, *d* = –1.106).

In the transverse plane, forefoot landing induced subtle but consistent increases in internal rotation at the hip and ankle joints. The hip showed significantly greater internal rotation during early (0.0%–4.2%, *p* = 0.0397, *d* = 0.763) and mid-to-late stance phases (67.0%–84.0%, *p* = 0.0012, *d* = 1.212). No significant differences were found at the knee. At the ankle, a brief increase in internal rotation was observed in forefoot landing during initial contact (0.0%–6.2%, *p* = 0.0281, *d* = 0.993).

### 3.3 GRF, GRF inclination angle and shank inclination angle

**Figure 6:**
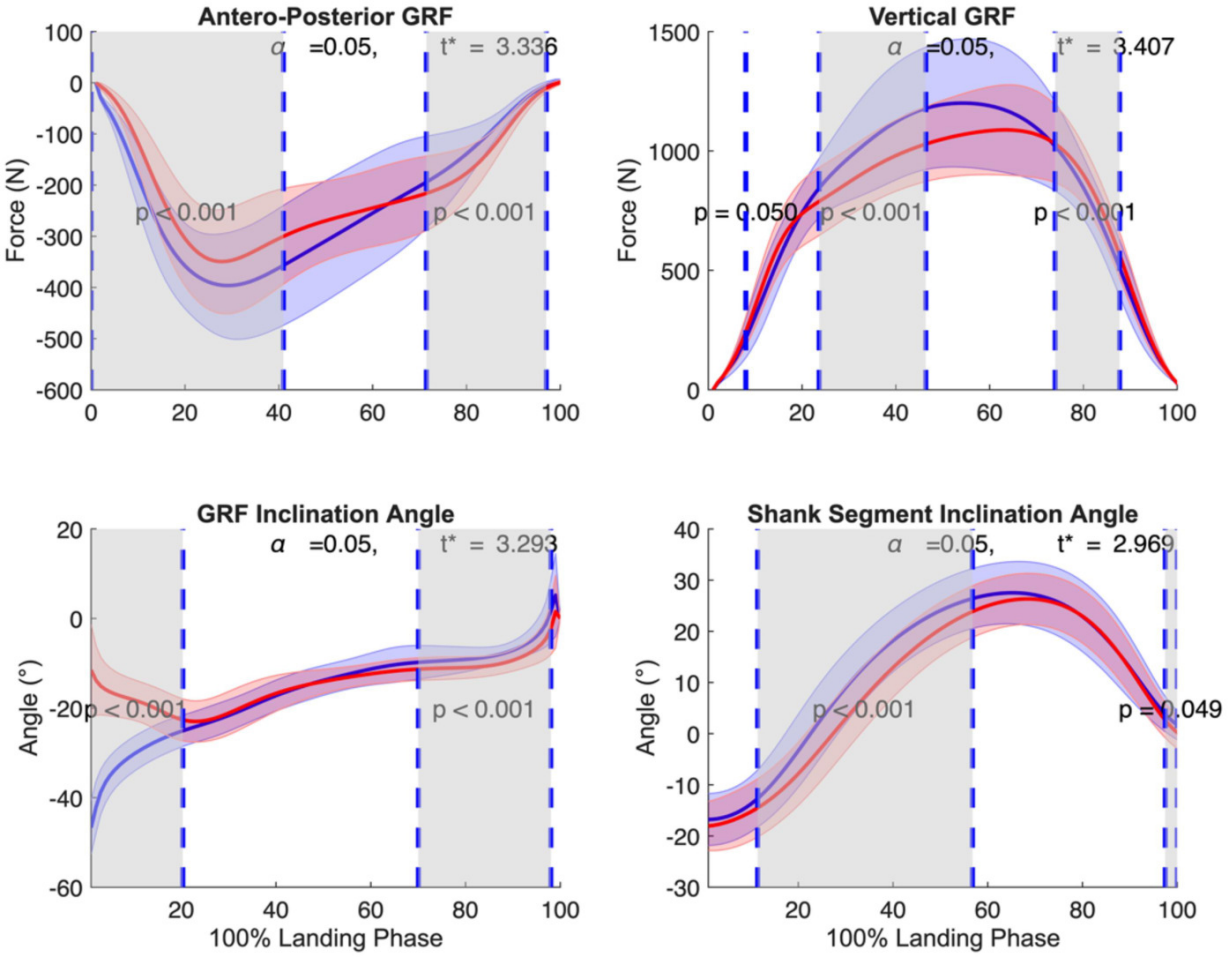
Mean ± SD and SPM t-values of GRF in posterior and vertical direction, GRF inclination angle, and shank segment inclination angle comparing forefoot landing (blue) and rearfoot landing (red) from initial ground contact to takeoff (normalized to 100% landing phase) in 23 subjects. The significance level was set at *p* = 0.05. Statistical differences are highlighted in grey-shaded regions, indicating *p* < 0.05.

Ground reaction force (GRF) analysis revealed distinct patterns between landing strategies (Figure 6). In the anterior-posterior (X) direction, forefoot landing exhibited significantly greater posterior GRF during early stance (0% to 41%, *p* < 0.001, *d* = 1.43) and significantly decreased posterior GRF during late stance (71% to 97%, *p* < 0.001, *d* = 1.84).

In the vertical direction, forefoot landing showed smaller vertical GRF at initial contact (8%, *p* = 0.050, *d* = 0.70), greater vertical GRF during mid-stance (24% to 46%, *p* < 0.001, *d* = 0.75), and smaller vertical GRF again during late stance (74% to 88%, *p* < 0.001, *d* = 1.27).

In the GRF angle, forefoot landing demonstrated a more posteriorly inclined GRF angle during early stance (0% to 20%, *p* < 0.001, *d* = 3.93), and a more anteriorly inclined GRF angle during late stance (70% to 98%, *p* < 0.001, *d* = 1.20).

At the shank segment, forefoot landing resulted in a more anteriorly inclined shank during mid-stance (11% to 57%, *p* < 0.001, *d* = 1.31) and terminal stance (97% to 100%, *p* = 0.049, *d* = 0.74).

### 3.4 Ankle variables at initial contact

Table 1 shows the ankle joint kinematics and kinetics at initial contact for both landing strategies. In the sagittal plane, forefoot landing exhibited greater plantarﬂexion (−26.02° ± 5.65° vs. 18.81° ± 5.61°, *p* < 0.001, *d* = −7.963) and greater plantarﬂexion moment (−0.21 ± 0.06 vs. 0.03 ± 0.03 Nm/kg, *p* < 0.001, d = −5.060). In the transverse plane, forefoot landing showed greater internal rotation (3.67° ± 4.59° vs. −3.32° ± 4.06°, *p* < 0.001, *d* = 0.185) and internal rotation moment (0.03 ± 0.02 vs. 0.02 ± 0.01 Nm/kg, *p* = 0.017, *d* = 1.177). In the frontal plane, while ankle angles were similar (3.45° ± 5.30° vs. 2.65° ± 3.06°, *p* = 0.566, *d* = 1.613), forefoot landing demonstrated greater inversion moment (0.02 ± 0.03 vs. −0.01 ± 0.02 Nm/kg, *p* = 0.002, *d* = 0.632).

**Table 1:** Ankle joint kinematics and kinetics at initial contact.

Variables	Forefoot	Rearfoot	*p*-value	t	d
Sagittal plane					
Dorsiﬂexion/Plantarﬂexion (°)	−26.02 (5.65)	18.81 (5.61)	< 0.001 *	−18.86	−7.963
Ankle moment (Nm/kg)	−0.21 (0.06)	0.03 (0.03)	< 0.001 *	−18.86	−5.060
Frontal plane					
Eversion/Inversion (°)	3.45 (5.30)	2.65 (3.06)	0.56582	3.61	0.185
Ankle moment (Nm/kg)	0.02 (0.03)	−0.01 (0.02)	0.002 *	3.61	1.177
Transverse plane					
External/Internal rotation (°)	3.67 (4.59)	−3.32 (4.06)	< 0.001 *	2.58	1.613
Ankle moment (Nm/kg)	0.03 (0.02)	0.02 (0.01)	0.017 *	2.58	0.632

* indicates significant difference (*p* < 0.05).

### 3.5 Jump height, stance time and approach speed

Performance variables during the stop-jumping task are presented in Table 2. No significant difference was observed in jump height between forefoot (49.51 ± 9.12 cm) and rearfoot landings (50.07 ± 9.06 cm, *p* = 0.34, *d* = 0.061). Stance time was significantly shorter in forefoot landing compared to rearfoot landing (396.75 ± 100.27 ms vs. 433.48 ± 62.67 ms, *p* = 0.01, *d* = 0.768). No significant difference was observed in approach speed between forefoot and rearfoot landings (2.14 ± 0.31 vs. 2.14 ± 0.34 m/s, *p* = 0.85, *d* = 0.163).

**Table 2:** Performance variables during stop-jumping task.

Variables	Forefoot landing	Rearfoot landing	*p*-value	d
Jump height (cm)	49.51 (9.12)	50.07 (9.06)	0.34	0.061
Stance time (ms)	396.75 (100.27)	433.48 (62.67)	0.01*	0.768
Approach speed (m/s)	2.14 (0.31)	2.14 (0.34)	0.85	0.163

Values are presented as mean (SD).

* indicates significant difference (*p* < 0.05).

## 4 Discussion

The present study examined the effects of forefoot vs. rearfoot landing strategies on lower limb biomechanics and performance during the stop-jumping task. The results revealed three key findings: (1) forefoot landing exhibited significantly greater ankle internal rotation at initial contact and during early landing phase; (2) forefoot landing showed distinct GRF characteristics, including greater posterior GRF and more posteriorly inclined GRF angle during early stance phase; and (3) forefoot landing demonstrated comparable jump height but shorter stance time compared to rearfoot landing.

Our analysis of knee kinematics (Figure 4) revealed no significant differences in knee ﬂexion angle between landing conditions during initial contact and early stance (0%–15%), a period critical for ACL injury risk (14, 30). While our first hypothesis predicted that forefoot landing would reduce ACL injury risk factors, we found mixed support for this prediction. The direct knee kinematics and kinetics were similar between conditions, suggesting that both landing strategies maintained comparable knee joint control. This finding differs from previous landing technique comparisons (16) but aligns with Walsh et al. (38), suggesting that knee kinematics during stop-jumping tasks may be more inﬂuenced by performance demands than landing strategy selection. However, as explored in our GRF analysis, other biomechanical factors may contribute to ACL protection.

Our analysis of ground reaction forces and segment orientations revealed distinct patterns between landing strategies (Figure 6). Forefoot landing showed larger posterior GRF components during early stance and a more posteriorly inclined GRF angle, consistent with previous findings by Uno et al. (26). These biomechanical features partially support our first hypothesis by potentially reducing ACL loading through counteracting anterior tibial translation. The posterior GRF may create a posterior shear force at the tibia that could counteract the anterior shear force generated by quadriceps contraction, which has been proposed as one mechanism contributing to ACL strain. Shin et al. (39) demonstrated through computational modeling that increasing posterior force from 0% to 30% of vertical impact force reduced peak ACL strain by up to 91%. This protective effect occurs during the critical first 40 ms after impact, precisely when ACL strain peaks and before neuromuscular protective responses can fully engage. While Boden et al. (20) have suggested that excessive compressive forces leading to anterior tibial translation may be more critical in ACL injury than previously thought, the relative contribution of different loading mechanisms remains debated. The relationship between our observed GRF patterns and ACL loading cannot be directly confirmed without *in vivo* measurements, but the increased posterior GRF component during early stance in forefoot landing may provide a biomechanical advantage for ACL protection.

Our findings regarding ankle kinematics (Table 1, Figure 4) supported our second hypothesis that forefoot landing would increase lateral ankle sprain risk. At initial contact, forefoot landing exhibited greater ankle internal rotation (3.67° vs. −3.32°) and plantarﬂexion (−26.02° vs. 18.81°). During early stance, SPM analysis showed significant differences in ankle internal rotation (0%–31%) and inversion (16% to 29%). These altered ankle positions are concerning given previous research on actual ankle sprain incidents. Analysis of tennis injuries has shown that ankle sprains typically involve sudden inversion combined with internal rotation, with even small initial joint misalignments potentially leading to injury (40). This mechanism has been further confirmed through laboratory capture of an accidental sprain, which demonstrated how rapid increases in inversion and internal rotation can progress to injury (41). The increased ankle internal rotation and inversion we observed during forefoot landing may therefore create a mechanically disadvantageous position that increases lateral ankle ligament stress and potentially places the athlete at higher risk for lateral ankle sprains, particularly during rapid deceleration movements like the stop-jumping task.

Our analysis revealed that forefoot landing achieved comparable jump heights with significantly shorter stance time (Table 2), a finding particularly relevant for sport performance where quick execution can create tactical advantages. This partially supports our third hypothesis, which predicted that forefoot landing would enhance performance outcomes including jump height and reduce stance time through more effective utilization of the stretch-shortening cycle. While we observed the hypothesized reduction in stance time, we did not find the expected improvement in jump height. Interestingly, our findings appear to contrast with previous studies of a forefoot-based soft landing technique, which involves greater knee and hip ﬂexion during impact that showed longer stance time (15, 42). This discrepancy likely stems from different task demands and movement priorities. In prior studies of soft landings, participants were typically instructed to prioritize impact attenuation by increasing knee and hip ﬂexion to attenuate impact forces, thereby prolonging the deceleration phase and reducing the peak GRF. Consequently, these studies generally reported longer stance time. In contrast, the stop-jumping task in our study required participants to perform with maximal performance—that is, to decelerate and take off as quickly and as powerfully as possible. This performance-oriented task demand likely encouraged participants to adopt a more reactive landing pattern, relying on the elastic energy storage and release of the plantar ﬂexor muscles to achieve shorter contact durations and higher jump efficiency (27, 28). Thus, differing task objectives—impact attenuation vs. performance maximization—can substantially alter the temporal and mechanical characteristics of landing. Our results indicate that the forefoot landing strategy effectively shortened stance duration while maintaining jump performance, suggesting that it may represent an advantageous approach for sport-specific scenarios requiring rapid response and high explosive power.

This study has several limitations. First, our exclusive focus on male subjects limits generalizability, particularly given that females have a higher incidence of noncontact ACL injuries than males (43–46). Second, despite implementing randomized trial order and standardized rest periods, the multiple maximum-effort trials required in this study may still have inﬂuenced some level of fatigue. Finally, our analysis was limited to the stop-jumping task; future research should examine these landing strategies across other high-risk movements common in sports, such as cutting maneuvers and single-leg landings.

Our study demonstrates that forefoot landing during stop-jumping tasks presents both advantages and risks. This strategy potentially reduces ACL injury through increased posterior GRF risk and improves performance through decreased stance time without compromising jump height. However, the increased ankle internal rotation and inversion observed during forefoot landing may elevate the risk of lateral ankle sprains. These findings have important practical implications for athletes and coaches. While forefoot landing may be advantageous for performance and ACL injury prevention, its implementation in training programs should be accompanied by specific emphasis on developing ankle joint stability and control. Future research should examine whether targeted ankle stabilization training can help athletes maintain the performance benefits of forefoot landing while minimizing ankle sprain risk, particularly across diverse populations and various sport-specific movements.

## Data Availability

The datasets presented in this article cannot be shared due to ethical restrictions and participant confidentiality agreements approved by the institutional review board. The data contain sensitive human motion capture and force plate recordings that may potentially be identifiable. Requests to access the datasets should be directed to Tianchen Huang, htc455868817@gmail.com.
